# Capnography as an aid in localizing the phrenic nerve in brachial plexus surgery. Technical note

**DOI:** 10.1186/1749-7221-3-14

**Published:** 2008-05-22

**Authors:** Hemant Bhagat, Anil Agarwal, Manish S Sharma

**Affiliations:** 1 Department of Neuroanesthesia, All India Institute of Medical Sciences, New Delhi-110029, India; 2 Department of Neurosurgery, All India Institute of Medical Sciences, New Delhi-110029, India

## Abstract

**Background:**

To determine whether monitoring end- tidal Carbon Dioxide (capnography) can be used to reliably identify the phrenic nerve during the supraclavicular exploration for brachial plexus injury.

**Methods:**

Three consecutive patients with traction pan-brachial plexus injuries scheduled for neurotization were evaluated under an anesthetic protocol to allow intraoperative electrophysiology. Muscle relaxants were avoided, anaesthesia was induced with propofol and fentanyl and the airway was secured with an appropriate sized laryngeal mask airway. Routine monitoring included heart rate, noninvasive blood pressure, pulse oximetry and time capnography. The phrenic nerve was identified after blind bipolar electrical stimulation using a handheld bipolar nerve stimulator set at 2–4 mA. The capnographic wave form was observed by the neuroanesthetist and simultaneous diaphragmatic contraction was assessed by the surgical assistant. Both observers were blinded as to when the bipolar stimulating electrode was actually in use.

**Results:**

In all patients, the capnographic wave form revealed a notch at a stimulating amplitude of about 2–4 mA. This became progressively jagged with increasing current till diaphragmatic contraction could be palpated by the blinded surgical assistant at about 6–7 mA.

**Conclusion:**

Capnography is a sensitive intraoperative test for localizing the phrenic nerve during the supraclavicular approach to the brachial plexus.

## Background

Early surgical intervention after brachial plexus injury is the best predictor of a favourable functional outcome after a trial of conservative management. Electrodiagnostic studies like sensory evoked potentials (SEP), electromyography (EMG) and nerve compound action potentials (NCAPs) are performed intraoperatively to aid in monitoring, guiding, identifying and localizing nerve function.[1] Though these diagnostic modalities have contributed immensely to the improved surgical outcomes following brachial plexus repair, their use may prove cumbersome and prone to errors of interpretation. Direct observation of muscle belly contraction after nerve stimulation remains the gold standard to detect intact neuronal function.

Phrenic nerve identification is a key step during the supraclavicular approach for brachial plexus surgery. Capnography is a technique to record end-tidal carbon dioxide (ETCO_2_) and is one of the standards of monitoring in anesthetic care. The authors describe the use of capnography as an aid in the intraoperative localization of the phrenic nerve.

## Methods

Three adult patients with diagnosed traction panbrachial plexus lesions were scheduled for supra and infraclavicular exploration and neurotization of the suprascapular, axillary and musculocutaneous nerves.

The general anaesthetic technique was tailored to allow intraoperative electrophysiological techniques to guide the localization and repair of the injured nerves. Consequently, muscle relaxants were avoided. Anaesthesia was induced with propofol and fentanyl and the airway was secured with a laryngeal mask. Anaesthesia was maintained with a propofol infusion and intermittent boluses of fentanyl. Routine monitoring included heart rate, non-invasive blood pressure, pulse oximetry and time capnography.

Supraclavicular exploration was commenced in the supine position with the head extended and turned to the opposite side and the injured arm in an adducted position. The skin incision was extended inferiorly over the lower 1/3^rd ^of the posterior border of the clavicular head of the sternocleidomastoid and then curved laterally over the medial 2/3^rd ^of the superior surface of the clavicle. The platysma was incised and the supraclavicular pad of fat was dissected sharply under the microscope away from the carotid sheath and the subclavian vein and retracted posterolaterally. The omohyoid bellies were then identified and their common tendinous insertion was divided between ligatures. The Scalenus anticus was then sought as the musculofascial structure behind the phrenic nerve. In view of the extensive scarring, the visual identification of the phrenic nerve was not possible at first. Hence, blind bipolar electrical stimulation using a handheld bipolar nerve stimulator was used to localize the same by eliciting diaphragmatic contraction. The nerve stimulator was initially used at low amplitude (1 mA) and the capnographic wave form was observed. The changes in waveform were monitored by the neuroanesthetist as the stimulating current was gradually increased. Simultaneously, the presence of diaphragmatic contraction was judged by the surgical assistant with his hand placed over the patient's draped epigastrium. Both the neuroanesthetist and the surgical assistant were blinded as to when the bipolar stimulating electrode was actually in use. Once the phrenic nerve was approximately localized, sharp dissection was commenced to identify the same.

## Results

**Figure 1:**
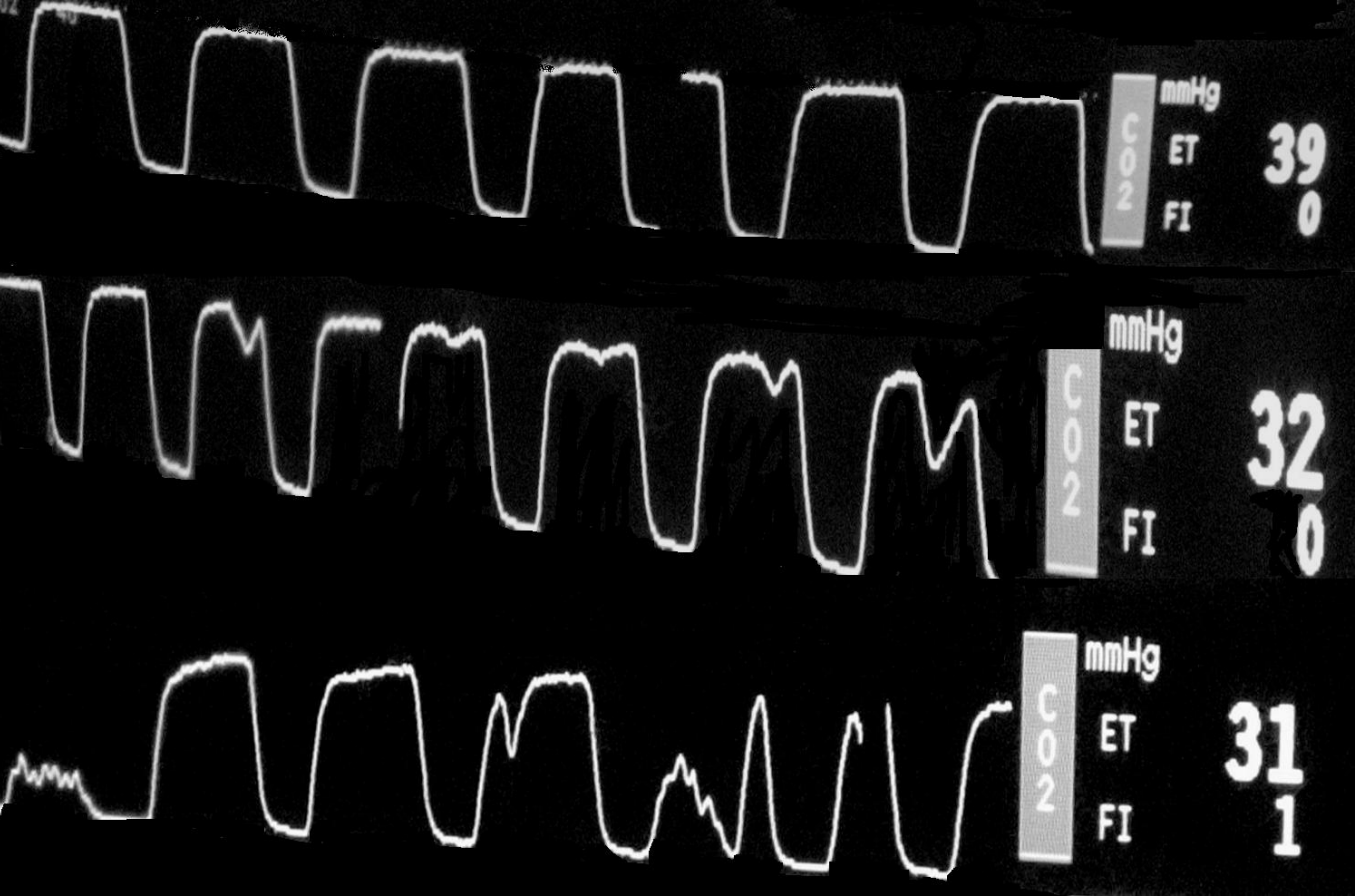
**Fused capnograms as seen on the patient monitor**. The top row is normal. After electrical stimulation, the middle row reveals progressive notching of the wave form (subclinical diaphragmatic contraction) which degenerates into frank spikes with increasing current corresponding to palpable diaphragmatic contractions. The progressive drop in end tidal CO_2 _from a baseline of 39 mm Hg to 31 mm Hg is noteworthy.

In all patients, the capnographic wave form revealed a notch at a low electrical stimulating current of about 2–4 mA. This became progressively jagged with increasing current strengths till diaphragmatic contraction could be palpated by the blinded surgical assistant at about twice the amplitude (6–7 mA). (Fig 1)

## Discussion

Brachial plexus lesions most frequently affect the supraclavicular region rather than the retroclavicular or infraclavicular levels.[2] Hence, the supraclavicular approach is the most commonly performed for traumatic brachial plexus repair. Intraplexal and extraplexal nerve-transfers are increasingly being utilized for brachial plexus reconstruction aimed at restoring elbow flexion and shoulder abduction.[3] Commonly used donor nerves are the thoracic intercostals, the medial pectoral, the phrenic and the spinal accessory nerves.

Intraoperative monitoring of nerve repair using electrodiagnostic techniques aids the surgeon in the dissection, identification and localization of nerves and also helps in assessing nerve function. Electrodiagnosis proves valuable, more so, in a setting of extensive fibrosis in the supraclavicular compartment frequently encountered after traction brachial plexus injuries. This makes identification of the Scalenus anticus, behind which the C5 and C6 nerve roots lie, very difficult especially when this key muscle is fibrosed and merges with the surrounding neuroma. The muscle is then indirectly identified as the tissue lying behind the phrenic nerve. The phrenic nerve is the only structure in the medial supraclavicular area which passes from lateral to medial. Thus, phrenic nerve identification is the crucial initial step in the supraclavicular approach for brachial plexus repair. Direct visualization may not be possible even under high magnification as the phrenic nerve too is often encased by scar tissue. Hence, blind stimulation using a hand held bipolar electrical stimulator and judging the contractile response of the diaphragm manually is a useful aid in initial localization before attempting scar tissue release with sharp dissection.

Other surgical techniques to identify the phrenic nerve include following the supraclavicular nerve proximally till the C4 root in order to identify the Phrenic nerve.[4] However, most brachial plexus surgeons prefer to use intraoperative electrical stimulation.

Monitoring phrenic nerve stimulation using lower chest wall electrodes may produce false-positive results due to co-activation of the brachial plexus.[5,6] Other possible technical problems include overstimulation, stimulus artifacts, electrical noise, and high recording electrode impedance which may diminish reliability and increase the duration of the procedure.[7]

On the other hand, capnography is a routine and mandatory anaesthetic monitoring device. In this study, a notch in the capnograph could be obtained at a lower stimulus intensity than palpable diaphragmatic contraction. Phrenic nerve stimulation in an anesthetized non-paralyzed patient produces sub-clinical diaphragmatic contraction which mimics inspiration. This produces a drop in ETC0_2 _which is reflected as a notch on the time capnograph. These observations were similar and reproducible in all the three patients. The authors could not come across any report in medical literature utilizing this attribute of capnography as an indicator of phrenic nerve stimulation in brachial plexus surgery in a non-paralysed patient. Electromyographic electrode placement to detect phrenic nerve activity may also be affected by concurrent stimulation of the other intraplexal nerves such as the thoracodorsal.[8] A notch in the capnogram, however, cannot be produced upon stimulation of the brachial plexus, thereby rendering this technique not only highly sensitive but also highly specific. The phrenic nerve also has a large number of motor axons and thus serves as an excellent donor nerve.[9] Capnography thus may help prevent inadvertent damage to the same by alerting the surgeon to its presence in difficult cases with extensive scarring.

## Conclusion

Capnography is a sensitive intraoperative test for localizing the phrenic nerve during the supraclavicular approach to the brachial plexus.

## Competing interests

*Financial competing interests*

In the past five years have you received reimbursements, fees, funding, or salary from an organization that may in any way gain or lose financially from the publication of this manuscript, either now or in the future? Is such an organization financing this manuscript? If so, please specify.

***No***.

Do you hold any stocks or shares in an organization that may in any way gain or lose financially from the publication of this manuscript, either now or in the future? If so, please specify.

***No***.

Do you hold or are you currently applying for any patents relating to the content of the manuscript? Have you received reimbursements, fees, funding, or salary from an organization that holds or has applied for patents relating to the content of the manuscript? If so, please specify.

***No***.

Do you have any other financial competing interests? If so, please specify.

***No***.

*Non-financial competing interests*

Are there any non-financial competing interests (political, personal, religious, ideological, academic, intellectual, commercial or any other) to declare in relation to this manuscript? If so, please specify. ***No***.

## Authors' contributions

HB made the original observation on the capnograph, was the blinded anesthetist and helped edit the manuscript. AA wrote the manuscript's first draft, carried out the literature search and was a blinded anesthetist. MSS conceived the concept, elucidated the methodology and edited the manuscript.
